# Mediation effect of stroke recurrence in the association between post‐stroke interleukin‐6 and functional disability

**DOI:** 10.1111/cns.14289

**Published:** 2023-06-08

**Authors:** Hong‐Qiu Gu, Kai‐Xuan Yang, Jie‐Jie Li, Jin‐Xi Lin, Jing Jing, Yun‐Yun Xiong, Xing‐Quan Zhao, Yi‐Long Wang, Li‐Ping Liu, Xia Meng, Yong Jiang, Hao Li, Yong‐Jun Wang, Zi‐Xiao Li

**Affiliations:** ^1^ China National Clinical Research Center for Neurological Diseases, Beijing Tiantan Hospital Capital Medical University Beijing China; ^2^ National Center for Healthcare Quality Management in Neurological Diseases, Beijing Tiantan Hospital Capital Medical University Beijing China; ^3^ Vascular Neurology, Department of Neurology, Beijing Tiantan Hospital Capital Medical University Beijing China; ^4^ Research Unit of Artificial Intelligence in Cerebrovascular Disease, Chinese Academy of Medical Sciences Beijing China; ^5^ Neuro‐Intensive Care Unit, Department of Neurology, Beijing Tiantan Hospital Capital Medical University Beijing China; ^6^ Chinese Institute for Brain Research Beijing China

**Keywords:** functional disability, interleukin‐6, mediation analysis, stoke recurrence

## Abstract

**Aim:**

Post‐stroke inflammation increases the risk of functional disability through enlarged cerebral infarct size directly and follow‐up stroke event indirectly. We aimed to use post‐stroke proinflammatory cytokine interleukin‐6 (IL‐6) as a marker of inflammatory burden and quantify post‐stroke inflammation's direct and indirect effect on functional disability.

**Methods:**

We analyzed patients with acute ischemic stroke admitted to 169 hospitals in the Third China National Stroke Registry. Blood samples were collected within 24 h of admission. Stroke recurrence and functional outcome measured by the modified Rankin scale (mRS) were assessed via face‐to‐face interviews at 3 months. Functional disability was defined as an mRS score ≥2. Mediation analyses under the counterfactual framework were performed to examine the potential causal chain in which stroke recurrence may mediate the relationship between IL‐6 and functional outcome.

**Results:**

Among the 7053 analyzed patients, the median (interquartile range [IQR]) NIHSS score was 3 (1–5), and the median (IQR) level of IL‐6 was 2.61 (1.60–4.73) pg/mL. Stroke recurrence was observed in 458 (6.5%) patients, and functional disability was seen in 1708 (24.2%) patients at the 90‐day follow‐up. Per stand deviation (4.26 pg/mL) increase in the concentration of IL‐6 was associated with an increased risk of stroke recurrence (adjusted odds ratio [aOR], 1.19; 95% CI, 1.09–1.29) and disability (aOR, 1.22; 95% CI, 1.15–1.30) within 90 days. Mediation analyses revealed that 18.72% (95% CI, 9.26%–28.18%) of the relationship between IL‐6 and functional disability was mediated by stroke recurrence.

**Conclusions:**

Stroke recurrence mediates less than 20% of the association between IL‐6 and functional outcome at 90 days among patients with acute ischemic stroke. In addition to typical secondary prevention strategies for preventing stroke recurrence, more attention should be paid to novel anti‐inflammatory therapy to improve functional outcomes directly.

## Graphical abstract

Stroke recurrence mediates less than 20% of the association between post‐stroke proinflammatory cytokine interleukin‐6 and functional outcome at 90 days among patients with acute ischemic stroke.

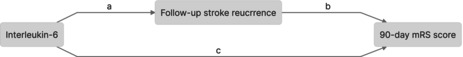

## 1 INTRODUCTION

Ischemic stroke, accounting for over 80% of strokes, is the largest contributor to the disease burden of China's healthcare system.^1^ A recent survey in China estimated that ischemic stroke's age‐ and sex‐standardized prevalence was 2.3% in 2020.^2^ With continuous efforts in the medical field and advancements in clinical practice, the 3 months recurrence of ischemic stroke has declined from 16.6% in 2007–2008 in the China National Stroke Registry (CNSR‐I) to less than 10% in 2015–2018 in the CNSR‐III.^3^, ^4^ However, the rate of unfavorable functional outcomes remains as high as approximately 30%.^5^

From the biological perspective, inflammation plays a critical role in the pathogenesis and prognosis of ischemic stroke, both acutely and chronically.^6^, ^7^ Interleukin‐6 (IL‐6) is a critical post‐stroke proinflammatory cytokine and a marker of inflammatory burden.^8^ Previous reports demonstrated that increased levels of IL‐6 were associated with the larger sizes of the brain lesions, thus resulting in poor functional outcomes directly.^9^, ^10^, ^11^ On the other hand, IL‐6 may contribute to the pathogenesis and progression of atherosclerosis, plaque rupture, platelet aggregation, and intravascular thrombosis, which increase the risk of following stroke events and result in further functional damage.^7^ Although prior studies reported associations between the higher concentration of IL‐6 and higher risks of stroke recurrence or functional impairment,^12^, ^13^, ^14^, ^15^, ^16^, ^17^, ^18^ none of them quantified the extent of functional impairment resulting from IL‐6 directly or resulting from the pathway of IL‐6–stroke recurrence–functional impairment.

Using data from the CNSR‐III study, we investigated to what extent, if any, stroke recurrence mediated the relationships between IL‐6 and functional disability at 90 days among patients with acute ischemic stroke and assessed the mediation effect in a series of sensitivity analyses.

## 2 METHODS

The study was approved by the ethics committee of Beijing Tiantan Hospital (KY2015–001‐01). Written informed consent was obtained from each participant or his/her representative before data collection. This report follows A Guideline for Reporting Mediation Analyses of Randomized Trials and Observational Studies reporting guideline.^19^

### 2.1 Study design and participants

We extracted data from the Third China National Stroke Registry (CNSR‐III), a large‐scale nationwide prospective registry of acute ischemic cerebrovascular events in China. Details for study design and patient identification of the CNSR‐III were reported elsewhere.^20^ In brief, patients with acute ischemic stroke or transient ischemic attack (TIA) occurred within 7 days and aged 18 years old or above were consecutively enrolled in this registry between August 2015 and March 2018. According to the World Health Organization criteria, acute ischemic stroke was diagnosed with confirmation by brain magnetic resonance imaging (MRI) or computed tomography (CT).^21^

For the current analysis, we included patients from 169 sites participating in the prespecified biomarker substudy and the imaging substudy. We excluded TIA patients and ischemic stroke patients with prior functional disabilities, patients who received reperfusion therapy (intravenous thrombolysis or thrombectomy), and patients with missing data on IL‐6 or 3‐month mRS scores.

### 2.2 Data collection

Baseline data were collected by trained research coordinators following a standard data collection protocol that the steering committee developed. Baseline data include demographics (sex, age, body mass index); National Institutes of Health Stroke Scale (NIHSS, range 0 to 42, with a higher score indicating greater stroke severity) score; pre‐stroke modified Rankin Scale (mRS, range 0 [no symptoms] to 6 [death]) score; systolic blood pressure (SBP); diastolic blood pressure (DBP); smoking status; and medical history, including prior stroke or TIA, hypertension, diabetes, dyslipidemia, coronary heart disease or myocardial infarction(CHD/MI), and atrial fibrillation.

Blood samples within 24 h of admission were collected. Plasma specimens were extracted, aliquoted, and transported through the cold chain to the central laboratory in Beijing Tiantan Hospital and stored at −80°C until tests were performed centrally and blindly. The concentration of IL‐6 was measured without technical repeat by using enzyme‐linked immunosorbent assay kits (catalog number: PHS600C, R&D Systems, Inc).

Imaging data were collected in Digital Imaging and Communications in Medicine (DICOM) format on discs and sent to the imaging research center of Beijing Tiantan Hospital. Acute infarction was diagnosed according to hyperintensity on diffusion‐weighted imaging and was further classified as single acute infarction (uninterrupted lesions visible in contiguous territories), multiple acute infarctions (more than one topographically distinct lesion), or watershed infarction.^22^ The etiology of each case was classified as large‐artery atherosclerosis (LAA), cardioembolism (CE), small‐artery occlusion (SAO), another determined cause or an undetermined by the classification of the Trial of Org 10,172 in Acute Stroke Treatment (TOAST) criteria.^23^

### 2.3 Patient follow‐up and outcomes

Trained research coordinators followed patients in face‐to‐face interviews based on a standardized interview protocol 90 days after symptom onset. The stroke recurrence was defined as a new neurological deficit lasting more than 24 h or rehospitalization with a diagnosis of ischemic stroke, intracerebral hemorrhage, or subarachnoid hemorrhage. The functional disability was defined as an mRS (range of 0[no disability] –6 [death]) score ≥ 2, as approximately half of the ischemic strokes were minor strokes in the CNSR‐III registry.

### 2.4 Statistical analysis

The study population was categorized according to quantiles of IL‐6 level. Normality for continuous variables was tested by Shapiro–Wilk tests and checked by Q‐Q plots as well. Baseline characteristics were described using means and standard deviations or medians and interquartiles for continuous variables and frequencies and percentages for categorical variables. Comparisons between groups were conducted using analysis of variance (ANOVA) or Kruskal–Wallis tests for continuous variables and chi‐square tests for categorical variables. We used a SAS macro called %ggBaseline to produce the baseline table automatically.^24^ We reported odds ratios (ORs) and 95% CIs based on logistic regression models to assess the associations between IL‐6 and stroke recurrence or disability at 90 days. IL‐6 level was treated as a categorical variable with Q1 as the reference group and then as a continuous variable with increments of one standard deviation in logistic models.

**FIGURE 1:**
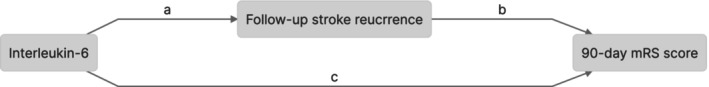
Illustration of mediation effect. mRS, modified Rankin Scale. Total effect = natural direct effect (c) + natural indirect effect (ab).

We used a directed acyclic graph to illustrate the association of IL‐6 with functional disability and follow‐up stroke recurrence (Figure 1). We performed causal mediation analysis under a counterfactual framework, in which a clear definition of the mediation effect was provided under a general framework.^25^, ^26^, ^27^ Under this framework, the total effect (TE) was divided into two parts measured as OR: the natural direct effect (NDE) and the natural indirect effect (NIE). The NDE represented the direct effect of IL‐6 on functional disability, while the NIE represented the effect of IL‐6 on functional disability via stroke recurrence. The mediation effect is measured by percentage mediated (PM), computed as NIE/TE*100% on a log‐transformed OR scale, which is the percentage of the TE that the mediator mediates.^28^ We fitted two logistic regression models to calculate the mediation effect. One is the mediator model, a multivariable logistic regression model for stroke recurrence (mediator) conditional on IL‐6 (exposure) and all study confounders. Another model is the outcome model, a multivariable logistic regression model for functional disability (outcome) conditional on IL‐6, stroke recurrence, and all study confounders. Based on the literature review and group discussion, confounders examined in the causal mediation analysis were determined, which included demographics (age, sex, and body mass index), NIHSS score at admission, smoking status, SBP, DBP, medical history (prior stroke/TIA, hypertension, diabetes mellitus, prior CHD/MI, and atrial fibrillation/flutter), image data (infarction pattern and infarction location) and stroke etiology where appropriate.

All hypothesis tests were 2‐tailed with a type‐1 error rate fixed at 5%. All statistical analyses were performed using SAS V.9.4 software (SAS Institute Inc.).

### 2.5 Sensitivity analyses

We performed a series of sensitivity analyses to test the robustness of our analysis. First, to avoid the competing risk with death, we re‐performed the mediation analysis after excluding patients who died before stroke recurrence. In addition, to check whether the mediation effect was affected by admission time, stroke severity, infarction pattern, infarction location, and subtype of ischemic stroke, we performed causal mediation analysis stratified by onset‐to‐door time (≤6 h, >6 h, or unknown), NIHSS score (>3, ≤3), infarction pattern, infarction location, and TOAST classification. Furthermore, we re‐assessed the association of IL‐6 with stroke recurrence and disability and re‐estimated the mediated effect of stroke recurrence on different scales of IL‐6 concentration, including original‐scale, log‐scale, and per standard deviation (SD) of log‐scale. Considering the impact of infarction volume, we provided a set of supplemental mediation analyses with the adjustment of infarction volume in the models as well.

## 3 RESULTS

We obtained data from 11,384 patients with acute ischemic stroke without baseline disability and without reperfusion treatment, excluding 3782 patients with TIA, mRS ≥ 2, or receiving reperfusion treatment from 15,166 admissions. After further exclusion of 4331 patients without blood or imaging samples, who had missing data on IL‐6 or lost to follow‐up, we finally included 7053 patients in the current analysis (Figure S1). Baseline characteristics were largely comparable between patients excluded and included (Table S1).

Of the 7053 patients analyzed, the age was 62.2 ± 11.3 years; 31.1% (*n* = 2193) of patients were women, and the median (IQR) NIHSS score was 3 (1‐5). Hypertension (62.8% [*n* = 4430]) was the most common disease history, followed by diabetes (24.5% [*n* = 1731]) and prior stroke/TIA (22.5% [*n* = 1584]). Nearly one‐half of the patients had a single infarction (47.0% [*n* = 3317]) and 56.9% [*n* = 4016] of patients had infarcts in the anterior circulation. For etiology classification, one‐quarter of patients were LAA (25.7% [*n* = 1810]), and another quarter was SAO (25.7% [*n* = 1813]) (Table 1).

**TABLE 1:** Baseline characteristics of patients by quartile of IL‐6 at admission.

Variables	Total (*N* = 7053)	Quartile 1 (*N* = 1762)	Quartile 2 (*N* = 1763)	Quartile 3 (*N* = 1764)	Quartile 4 (*N* = 1764)	*p* Value
IL‐6 level, pg/ml	2.61 (1.60–4.73)	<1.60	1.60–2.60	2.61–4.72	≥4.73	
Demographic						
Age	62.2 ± 11.3	58.5 ± 10.6	61.3 ± 10.7	63.2 ± 11.0	65.9 ± 11.5	<0.001
Women	2193 (31.1)	515 (29.2)	558 (31.7)	560 (31.7)	560 (31.7)	0.28
Smoking	2300 (32.6)	597 (33.9)	567 (32.2)	587 (33.3)	549 (31.1)	0.31
BMI	24.8 ± 3.3	24.6 ± 3.0	25.0 ± 3.2	24.8 ± 3.4	24.7 ± 3.7	<0.001
NIHSS score at admission	3.0 (1.0–5.0)	3.0 (1.0–5.0)	3.0 (1.0–5.0)	3.0 (2.0–6.0)	4.0 (2.0–7.0)	<0.001
SBP, mmHg	151.0 ± 22.5	150.7 ± 22.0	151.6 ± 22.6	151.7 ± 22.3	150.0 ± 23.1	0.07
DBP, mmHg	87.7 ± 13.2	88.9 ± 13.1	88.1 ± 12.9	87.8 ± 13.3	86.0 ± 13.4	<0.001
Medical history						
Prior stroke/TIA	1584 (22.5)	325 (18.4)	385 (21.8)	440 (24.9)	434 (24.6)	<0.001
Hypertension	4430 (62.8)	1056 (59.9)	1115 (63.2)	1133 (64.2)	1126 (63.8)	0.03
Diabetes mellitus	1731 (24.5)	349 (19.8)	459 (26.0)	494 (28.0)	429 (24.3)	<0.001
Dyslipidemia	567 (8.0)	119 (6.8)	139 (7.9)	173 (9.8)	136 (7.7)	0.008
Prior CHD/MI	696 (9.9)	116 (6.6)	170 (9.6)	184 (10.4)	226 (12.8)	<0.001
Atrial fib/flutter	441 (6.3)	42 (2.4)	71 (4.0)	116 (6.6)	212 (12.0)	<0.001
Infarction volume, mm^3^	1638 (708–6281)	1246 (616–3031)	1383 (650–4234)	1811 (725–6903)	3247 (989–15,390)	<0.001
Infarction pattern						
None	463 (6.6)	143 (8.1)	113 (6.4)	108 (6.1)	99 (5.6)	<0.001
Single infarction	3317 (47.0)	971 (55.1)	910 (51.6)	792 (44.9)	644 (36.5)	
Multiple infarction	3157 (44.8)	628 (35.6)	714 (40.5)	827 (46.9)	988 (56.0)	
Watershed infarction	116 (1.6)	20 (1.1)	26 (1.5)	37 (2.1)	33 (1.9)	
Infarction circulation						
None	463 (6.6)	143 (8.1)	113 (6.4)	108 (6.1)	99 (5.6)	<0.001
Anterior circulating infarction	4016 (56.9)	995 (56.5)	1002 (56.8)	1010 (57.3)	1009 (57.2)	
Posterior circulation infarction	2130 (30.2)	555 (31.5)	543 (30.8)	536 (30.4)	496 (28.1)	
Anterior and posterior circulatory infarction	444 (6.3)	69 (3.9)	105 (6.0)	110 (6.2)	160 (9.1)	
Stroke etiology						
LAA	1810 (25.7)	311 (17.7)	413 (23.4)	526 (29.8)	560 (31.7)	<0.001
CE	401 (5.7)	56 (3.2)	86 (4.9)	99 (5.6)	160 (9.1)	
SAO	1813 (25.7)	551 (31.3)	519 (29.4)	424 (24.0)	319 (18.1)	
Other	3029 (42.9)	844 (47.9)	745 (42.3)	715 (40.5)	725 (41.1)	

Abbreviations: BMI, body mass index; CE, cardioembolism; CHD, coronary artery disease; DBP, diastolic blood pressure; IL‐6, Interleukin 6; LAA, large‐artery atherosclerosis; MI, myocardial infarction; NIHSS, National Institutes of Health Stroke Scale; SAO, small‐artery occlusion; SBP, systolic blood pressure; and TIA, transient ischemic attack.

### 3.1 Baseline characteristics

The median (IQR) IL‐6 concentration was 2.61 (1.60–4.73) pg/mL. Figure S2 shows the distribution of IL‐6. Compared to patients in the lowest quartile of IL‐6, patients in the highest quartiles of IL‐6 were older (65.9 ± 11.5 vs. 58.5 ± 10.6, *p* < 0.001), had a severer stroke at admission (median[IQR] of NIHSS score: 4.0 (2.0–7.0) vs. 3.0 [1.0–5.0]; *p* < 0.001), and more vascular risk factors, including prior stroke/TIA (434 [24.6%] vs. 325 [18.4%], *p* < 0.001), hypertension (1126 [63.8%] vs. 1056 [59.9%], *p* = 0.03), diabetes (429 [24.3] vs. 349 [19.8], *p* < 0.001), dyslipidemia (136 [7.7%] vs. 119 [6.8%], *p* = 0.008), CHD/MI (226 [12.8%] vs. 116 [6.6%], *p* < 0.001), and atrial fibrillation (212 [12.0%] vs. 42 [2.4%], *p* < 0.001). In addition, more multiple infarctions (988 [56.0%] vs. 628 [35.6%], *p* < 0.001), simultaneous anterior and posterior circulatory infarctions (160 [9.1%] vs. 69 [3.9%], *p* < 0.001), and the LAA subtype of ischemic stroke (560 [31.7%] vs. 311 [17.7%], *p* < 0.001) were seen in the highest quartiles of IL‐6 (Table 1).

### 3.2 Associations of IL‐6 with stroke recurrence or functional disability

During the 90‐day follow‐up, stroke recurrence and functional disability were seen in 458 (6.5%) and 1708 (24.2%) patients, respectively. After adjusting for covariates, patients with the highest quartile of IL‐6 levels had a non‐significantly 27% higher risk of stroke recurrence (8.8% vs. 5.5%, adjusted OR [aOR], 1.27; 95% CI, 0.95–1.68) and a significantly 59% higher risk of disability (36.1% vs. 16.3%; aOR, 1.59; 95% CI, 1.32–1.91) at 90 days. We also treated IL‐6 concentration as a continuous variable. We found that per SD (4.26 pg/mL) increase in IL‐6 concentration was associated with a significantly 19% increased risk of stroke recurrence (aOR, 1.19; 95% CI, 1.09–1.29) and a significantly 22% increased risk of disability (aOR, 1.22; 95% CI, 1.15–1.30) at 90 days (Table 2).

**TABLE 2:** Associations of IL‐6 with stroke recurrence and functional disabilities at 90 days.

Outcomes	No of patients	Event (%)	Crude analysis		Adjusted analysis^a^	
			Crude OR (95% CI)	Crude *p*	Adjusted OR (95% CI)	Adjusted *p*
Stroke recurrence at 90 day	7053	458 (6.5)				
Quartile 1	1762	96 (5.5)	1.00 (Reference)		1.00 (Reference)	
Quartile 2	1763	101 (5.7)	1.05 (0.79–1.41)	0.72	0.96 (0.72–1.28)	0.77
Quartile 3	1764	106 (6.0)	1.11 (0.83–1.47)	0.47	0.92 (0.69–1.23)	0.60
Quartile 4	1764	155 (8.8)	1.67 (1.28–2.18)	<0.001	1.27 (0.95–1.68)	0.10
Per SD			1.26 (1.17–1.36)	<0.001	1.19 (1.09–1.29)	<0.001
Disability at 90 day	7053	1708 (24.2)				
Quartile 1	1762	287 (16.3)	1.00 (Reference)		1.00 (Reference)	
Quartile 2	1763	360 (20.4)	1.32 (1.11–1.57)	0.002	1.16 (0.96–1.40)	0.12
Quartile 3	1764	424 (24.0)	1.63 (1.38–1.92)	<0.001	1.15 (0.95–1.38)	0.15
Quartile 4	1764	637 (36.1)	2.90 (2.48–3.41)	<0.001	1.59 (1.32–1.91)	<0.001
Per SD			1.45 (1.38–1.52)	<0.001	1.22 (1.15–1.30)	<0.001

Abbreviations: CI, confidence intervals; IL‐6, Interleukin 6; OR, odds ratio; SD, standard deviation.

^a^ Adjusted for demographics (age, sex, body mass index), National Institutes of Health Stroke Scale score at admission, smoking status, systolic blood pressure, diastolic blood pressure, medical history (prior stroke/transient ischemic attack, hypertension, diabetes mellitus, prior coronary artery disease/myocardial infarction, atrial fib/flutter), and image data (infarction pattern, infarction location) and stroke etiology.

### 3.3 Mediation analyses

Stroke recurrence before disability and disability without stroke recurrence was seen in 16.2% (*n* = 277) and 83.8% (*n* = 1431) of the 1708 functionally disabled patients, respectively. Table 3 shows the total, direct associations, and indirect associations of IL‐6 with functional disability. The indirect effect of IL‐6 via follow‐up stroke recurrence implied a 4% increased risk of functional disability (aOR 1.04; 95% CI, 1.02–1.06) would be observed on average. The mediated proportion of the association between IL‐6 and functional disability by stroke recurrence was 18.72% (95% CI, 9.26%–28.18%) in the adjusted model. In addition, we excluded patients who died before stroke recurrence to avoid competing risk, and results showed a comparable mediation effect of stroke recurrence, with a mediated percentage of 23.16% (95% CI, 11.95%–34.37%).

**TABLE 3:** Associations between per SD of IL‐6 and 90‐day disability mediated by follow‐up stroke recurrence.

Effect	Unadjusted analysis		Adjusted analysis^a^	
	Estimate (95% CI)	*p*	Estimate (95% CI)	*p*
Of 7053 patients				
Total effect (TE), odds ratio	1.49 (1.41–1.57)	<0.001	1.25 (1.17–1.33)	<0.001
Natural direct effect (NDE), odds ratio	1.42 (1.35–1.50)	<0.001	1.20 (1.13–1.27)	<0.001
Natural indirect effect (NIE), odds ratio	1.05 (1.03–1.07)	<0.001	1.04 (1.02–1.06)	0.003
Percentage mediated (PM)	13.78 (8.98–18.57)	<0.001	18.72 (9.26–28.18)	<0.001
Of 7006 patients^b^				
Total effect (TE), odds ratio	1.45 (1.37–1.53)	<0.001	1.22 (1.14–1.30)	<0.001
Natural direct effect (NDE), odds ratio	1.38 (1.31–1.45)	<0.001	1.17 (1.09–1.24)	<0.001
Natural indirect effect (NIE), odds ratio	1.05 (1.03–1.07)	<0.001	1.04 (1.02–1.06)	<0.001
Percentage mediated (PM)	16.00 (10.71–21.28)	<0.001	23.16 (11.95–34.37)	<0.001

Abbreviations: IL‐6, Interleukin 6; mRS, modified Ranking Scale; SD, standard deviation.

^a^ Adjusted for demographics (age, sex, body mass index), National Institutes of Health Stroke Scale score at admission, smoking status, systolic blood pressure, diastolic blood pressure, medical history (prior stroke/transient ischemic attack, hypertension, diabetes mellitus, prior coronary artery disease/myocardial infarction, atrial fib/flutter), and image data (infarction pattern, infarction location) and stroke etiology.

^b^ With 47 death without stroke recurrence were excluded.

### 3.4 Sensitivity analyses

**FIGURE 2:**
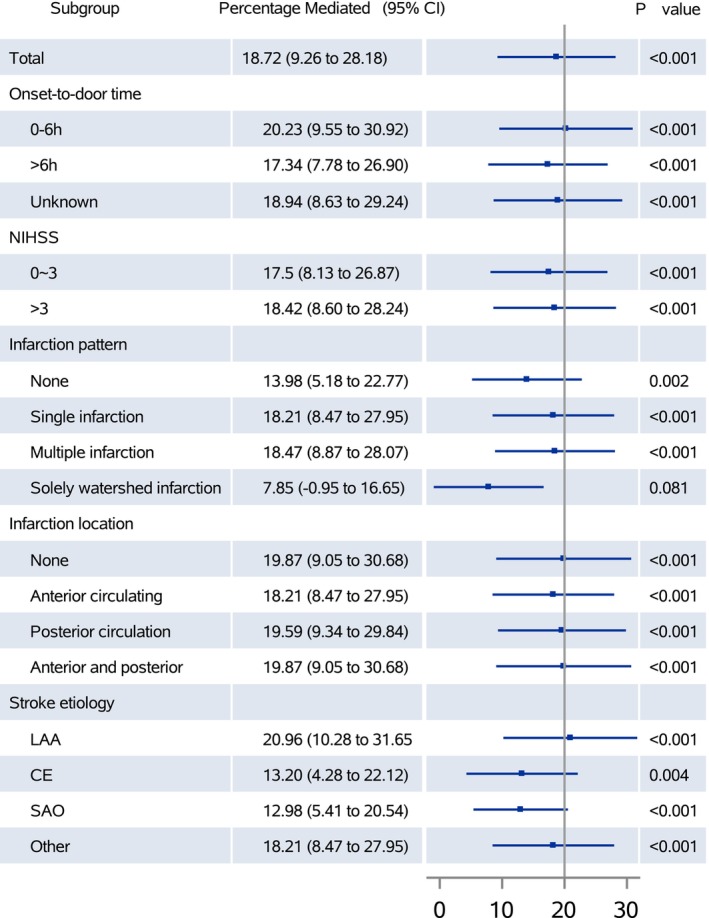
Causal mediation analysis stratified by prespecified subgroup. CE, cardioembolism; LAA, large‐artery atherosclerosis; NIHSS, the National Institutes of Health Stroke Scale; SAO, small‐artery occlusion.

Subgroup analysis revealed no heterogeneity in the major subgroup of admission time, NIHSS score at admission, infarction pattern, infarction location, and etiological subtype (Figure 2). Sensitivity analyses on different scales of IL‐6 concentrations showed similar results with previous analyses that IL‐6 was positively associated with the risk of stroke recurrence and functional disability (Table S2). The mediation analyses on different scales of IL‐6 concentration were also consistent with the primary analysis (percentage mediated, original‐scale: 17.47% [95% CI, 8.66%–26.28%]; log‐scale: 20.44% [95% CI, 8.22%–32.66%]; and per SD of log‐scale: 17.60% [95% CI, 6.69%–28.51%]) (Table 4). In addition, we additionally adjusted for infarction volume based on the primary mediation analyses, and the results showed a consistent conclusion (percentage mediated: 19.11% [95% CI, 5.49%–8.35%] for complete case analysis of infarction volume, 18.77% [95% CI, 8.13%–29.41%] for multiple imputation analysis of infarction volume) (Table S3).

**TABLE 4:** Association of IL‐6 with 90‐day disability mediated by follow‐up stroke recurrence on different sclaes of IL‐6.

Variables	Unadjusted analysis		Adjusted analysis^a^	
	Estimate (95% CI)	*p*	Estimate (95% CI)	*p*
Original‐scale of IL‐6				
Odds ratio total effect	1.09 (1.08–1.11)	<0.001	1.05 (1.04–1.07)	<0.001
Odds ratio natural direct effect (NDE)	1.08 (1.07–1.10)	<0.001	1.04 (1.03–1.06)	<0.001
Odds ratio natural indirect effect (NIE)	1.01 (1.01–1.01)	<0.001	1.01 (1.00–1.01)	<0.001
Percentage mediated	12.09 (7.80–16.37)	<0.001	17.47 (8.66–26.28)	<0.001
Log‐scale of IL‐6				
Odds ratio total effect	3.54 (2.96–4.13)	<0.001	1.82 (1.47–2.18)	<0.001
Odds ratio natural direct effect (NDE)	3.09 (2.61–3.57)	<0.001	1.65 (1.35–1.96)	<0.001
Odds ratio natural indirect effect (NIE)	1.15 (1.08–1.21)	<0.001	1.10 (1.03–1.17)	0.005
Percentage mediated	17.88 (11.30–24.46)	<0.001	20.44 (8.22–32.66)	<0.001
Per SD of log‐scale of IL‐6				
Odds ratio total effect	1.58 (1.48–1.67)	<0.001	1.24 (1.16–1.33)	<0.001
Odds ratio natural direct effect (NDE)	1.5 (1.42–1.59)	<0.001	1.20 (1.12–1.28)	<0.001
Odds ratio natural indirect effect (NIE)	1.05 (1.03–1.07)	<0.001	1.04 (1.01–1.06)	0.004
Percentage mediated	13.16 (8.18–18.14)	<0.001	17.60 (6.69–28.51)	0.002

^a^ Adjusted for demographics (age, sex, body mass index), National Institutes of Health Stroke Scale score at admission, smoking status, systolic blood pressure, diastolic blood pressure, medical history (prior stroke/transient ischemic attack, hypertension, diabetes mellitus, prior coronary artery disease/myocardial infarction, atrial fib/flutter), and image data (infarction pattern, infarction location) and stroke etiology.

Abbreviations: IL‐6, interleukin 6; mRS, modified Ranking Scale.

## 4 DISCUSSION

In this multicenter cohort study, mediation analyses revealed that less than 20% of the association between post‐stroke proinflammatory cytokine IL‐6 and functional disability at 90 days was mediated by stroke recurrence for patients with acute ischemic stroke, which indicates that more attention should be paid to the direct effect of post‐stroke inflammation associated functional disability.

Several previous studies have reported associations between IL‐6 and stroke recurrence^12^, ^13^, ^14^, ^15^ as well as associations between IL‐6 and functional disability independent of conventional risk factors among ischemic stroke patients.^16^, ^17^, ^18^ Results from Mendelian randomization analyses provided evidence for a causal effect of IL‐6 signaling on ischemic stroke as well.^29^, ^30^ In concordance with these previous reports, our study revealed that IL‐6 concentrations were positively associated with stroke recurrence and functional disability as well. These data from clinical practices provide a clue for our hypotheses. Our previous report also showed that follow‐up stroke recurrence mediated less than 20% effect of another proinflammatory cytokine high‐sensitivity C‐reactive protein on functional disability from a different pathway.^31^

In addition, evidence from biological research also provides a solid foundation for our hypothesis. Ischemic events cause neuroinflammation and the release of inflammatory cytokines from immune cells in brain tissues. IL‐6 is a well‐characterized proinflammatory cytokine that provokes and aggravates an inflammatory response after stroke.^32^ Although the pathophysiology of functional damage after a stroke is a complex cycle of interconnected molecular and cellular mechanisms,^33^ reports indicated that an increase in the concentration of IL‐6 in serum following a stroke increases injury by contributing to cell death and blood–brain barrier disruption.^6^, ^34^, ^35^ Other reports also suggested that IL‐6 plays an important role in cerebral ischemia not only as a mediator of the inflammatory process in the acute phase of stroke but also has chronic effects that might contribute to the formation and maturation of atherosclerotic plaques, which increase the risk of stroke recurrence and then resulted in functional damage as a neurotrophic factor in the late phase of the development of cerebral ischemia.^7^, ^33^ This was also consistent with the results revealed from our sensitivity analysis that LAA had a higher percentage of mediation (21%) by stroke recurrence than CE and SAO (13%).

Currently, the typical secondary prevention strategy for stroke is focused on antiplatelet, hypertension, diabetes, and dyslipidemia, while anti‐inflammation is usually ignored. Our analysis revealed that the indirect effect of post‐stroke proinflammatory cytokine IL‐6 on functional disability through stroke recurrence is less than 20%, which means that over 80% of functional damage results from the pathway of IL‐6 to functional disability without stroke recurrence. Therefore, anti‐inflammatory therapy and new therapies targeting poststroke inflammation, that is, cytokine inhibition, should be given more attention.^36^ Several previous studies have shown the potential benefits of targeted cytokine antagonist therapies for atherothrombosis.^37^, ^38^ A double‐blind, randomized, placebo‐controlled, phase 2 trial also showed that IL‐6 inhibition with ziltivekimab markedly reduced biomarkers of inflammation and thrombosis relevant to atherosclerosis in patients with advanced chronic renal disease.^39^ Whether the benefits of ziltivekimab for patients with ischemic stroke exist needs further studies. Other inhibitions, specific inhibition of the trans‐signaling pathway of IL‐6 using, for example, soluble glycoprotein 130 could be promising therapeutic tools in future stroke research.^40^

This study has several limitations. First, 38.0% (*n* = 4331) of 11,384 patients were excluded from this analysis because of no biomarker or imaging samples or missing data on IL‐6 or 90‐day follow‐up mRS, which may introduce selection bias. However, 95% (*n* = 4110) of the 4331 patients were excluded because they enrolled from a site not participating in the biomarker and imaging substudy. In addition, baseline characteristics between included and excluded patients were largely comparable. Therefore, we believe that the selection bias would be minimized in this study. Second, patients were recruited up to 7 days after stroke onset, which may introduce heterogeneity because of the admission time. However, over 50% of the blood sample were collected within 55 h after symptom onset, which would reduce the heterogeneity. In addition, subgroup analysis by admission time showed similar mediation effects. Third, during hospitalization, infections may affect the level of IL‐6 and the estimation of the mediation effect. However, the rate of pulmonary infection was only 4.9% (*n* = 343), and urinary system infection was only 1.3% (*n* = 91) among the included patients. In addition, we could not precisely judge whether these infections occurred before or after blood samples were taken. Fourth, the concentration of IL‐6 had a skewed distribution, which may affect the estimations of the mediation effect. However, sensitivity analyses on different scales of IL‐6 revealed stable and reliable results. Fifth, although potential covariates were carefully selected and adjusted for in the models when assessing the mediating effect, other unmeasured confounders may still exist. Finally, we exclude patients with prior functional disability and patients who received reperfusion therapy. Therefore, our conclusions may not be generalized to these populations.

## 5 CONCLUSIONS

In this multi‐center cohort study, we found that the association between post‐stroke proinflammatory cytokine IL‐6 and functional disability at 90 days in patients with ischemic stroke was only partially mediated by follow‐up stroke recurrence. In addition to the current strategy of secondary stroke prevention, novel anti‐inflammatory therapy should be given more attention to improve functional outcomes.

## AUTHOR CONTRIBUTIONS

Z‐XL and H‐QG conceived the study; H‐QG wrote the first draft of the manuscript; H‐QG and K‐XY performed the statistical analysis; J‐JL, J‐XL, JJ, XM and YJ coordinated patient recruitment and data collection; All authors contributed to the interpretation of the results. All authors reviewed and edited the manuscript and approved its final version.

## FUNDING INFORMATION

This work was supported by grants from the Beijing Hospitals Authority (QML20210501), Beijing Natural Science Foundation (Z200016), the National Natural Science Foundation of China (81870905, U20A20358), and the Beijing Municipal Science & Technology Commission (Z181100001818001).

## CONFLICT OF INTEREST STATEMENT

None.

## Supporting information


Appendix S1

## Data Availability

The data that support the findings of this study are available on request from the corresponding author.
