# Supplementary material for: Mediation effect of stroke recurrence in the association between post‐stroke interleukin‐6 and functional disability
Source: CNS Neurosci Ther. 2023 Jun 8;29(11):3579–87. doi: 10.1111/cns.14289 (PMC10580327; doi:10.1111/cns.14289)
Supplement: Supplementary file 1 — Appendix S1 [file CNS-29-3579-s001.docx]

**Supplementary Online Content**

**Mediation Effect of Stroke Recurrence in the Association between Post-stroke Interleukin-6 and Functional Disability**

**Table S1.** Comparison between included and excluded patients.

**Table S2.** Association between IL-6 and stroke recurrence and functional outcome at 90-day on different scales of IL-6.

**Table S3.** Associations between per SD of IL-6 and 90-day disability mediated by follow-up stroke recurrence, with models additionally adjusted for infarction volume.

**Figure S1**. Study flowchart

Abbreviations: IL-6 indicates Interleukin 6; mRS, modified Rankin Scale.

**Figure S2.** Distribution of IL-6 on different scales

**Table S1.** Comparison between included and excluded patients

| Variables | Total (N=11384 [100%]) | Include population (N=7053 [62.0%]) | Exclude population (N=4331 [38.0%]) | ASD/HL estimator (%) |
| --- | --- | --- | --- | --- |
| Demographic |  |  |  |  |
| Age |  |  |  |  |
| Mean±SD | 62.0±11.3 | 62.2±11.3 | 61.7±11.4 | 4.4 |
| Median (IQR) | 62.0 (54.0–70.0) | 62.0 (54.0–70.0) | 62.0 (54.0–69.0) | -1.0 |
| Female | 3556 (31.2) | 2193 (31.1) | 1363 (31.5) | 0.9 |
| Smoking | 3661 (32.2) | 2300 (32.6) | 1361 (31.4) | 2.6 |
| BMI | 24.7±3.3 | 24.8±3.3 | 24.7±3.2 | 3.1 |
| NIHSS score at admission |  |  |  |  |
| Mean±SD | 3.9±3.7 | 3.9±3.6 | 3.9±3.8 | 0.0 |
| Median (IQR) | 3.0 (1.0–5.0) | 3.0 (1.0–5.0) | 3.0 (1.0–5.0) |  |
| Blood pressure at admission |  |  |  |  |
| SBP | 150.6±22.2 | 151.0±22.5 | 150.0±21.7 | 4.5 |
| DBP | 87.7±13.1 | 87.7±13.2 | 87.6±13.0 | 0.8 |
| Medical history |  |  |  |  |
| Prior Stroke/TIA | 2507 (22.0) | 1584 (22.5) | 923 (21.3) | 2.9 |
| Hypertension | 7108 (62.4) | 4430 (62.8) | 2678 (61.8) | 2.1 |
| Diabetes mellitus | 2685 (23.6) | 1731 (24.5) | 954 (22.0) | 5.9 |
| Dyslipidemia | 862 (7.6) | 567 (8.0) | 295 (6.8) | 4.6 |
| Prior CHD/MI | 1133 (10.0) | 696 (9.9) | 437 (10.1) | 0.7 |
| Atrial fib/flutter | 679 (6.0) | 441 (6.3) | 238 (5.5) | 3.4 |

Abbreviations: NIHSS, National Institutes of Health Stroke Scale; IL-6 indicates Interleukin 6; SBP, systolic blood pressure; DBP, diastolic blood pressure; TIA, transient ischemic attack; CHD, coronary artery disease; MI, myocardial infarction; LAA, large-artery atherosclerosis; CE, cardioembolism; SAO, small-artery occlusion; ASD, absolute standard difference, an ASD > 10% means a clinically meaningful difference.

**Table S2.** Association between IL-6 and stroke recurrence and functional outcome at 90-day on different scales of IL-6.

| **Outcomes** | **Crude OR (95% CI)** | **Crude P** | **Adjusted OR (95% CI)^a^** | **Adjusted P** |
| --- | --- | --- | --- | --- |
| Stroke recurrence at 90 day |  |  |  |  |
| Original scale | 1.05 (1.04–1.07) | <.001 | 1.04 (1.02–1.06) | <.001 |
| Log scale | 1.97 (1.53–2.54) | <.001 | 1.54 (1.16–2.02) | 0.002 |
| per SD of log scale | 1.28 (1.17–1.40) | <.001 | 1.17 (1.06–1.29) | 0.002 |
| Disability at 90 day |  |  |  |  |
| Original scale | 1.09 (1.07–1.10) | <.001 | 1.05 (1.03–1.06) | <.001 |
| Log scale | 3.20 (2.75–3.72) | <.001 | 1.71 (1.43–2.04) | <.001 |
| per SD of log scale | 1.52 (1.44–1.61) | <.001 | 1.21 (1.14–1.29) | <.001 |

^a^adjusted for demographics (age, sex, body mass index), National Institutes of Health Stroke Scale score at admission, smoking status, systolic blood pressure, diastolic blood pressure, medical history (prior stroke/ transient ischemic attack, hypertension, diabetes mellitus, prior coronary artery disease/myocardial infarction, atrial fib/flutter), and image data (infarction pattern, infarction location) and stroke etiology.

**Table S3.** Associations between per SD of IL-6 and 90-day disability mediated by follow-up stroke recurrence, with models additionally adjusted for infarction volume.

| Effect | Adjusted analysis^*^ with the complete case of infarction volume | |  | Adjusted analysis^*^ with the multiple imputations of infarction volume † | |
| --- | --- | --- | --- | --- | --- |
|  | Estimate (95% CI) | P |  | Estimate (95% CI) | P |
| Total Effect (TE), Odds Ratio | 1.22 (1.14–1.30) | <.001 |  | 1.22 (1.14–1.30) | <.001 |
| Natural Direct Effect (NDE), Odds Ratio | 1.18 (1.10–1.25) | <.001 |  | 1.18 (1.10–1.26) | <.001 |
| Natural Indirect Effect (NIE), Odds Ratio | 1.04 (1.01–1.06) | <.001 |  | 1.04 (1.01–1.06) | 0.003 |
| Percentage Mediated (PM), % | 19.11 (5.49–8.35) | <.001 |  | 18.77 (8.13–29.41) | <.001 |

^*^Adjusted for demographics (age, sex, body mass index), National Institutes of Health Stroke Scale score at admission, smoking status, systolic blood pressure, diastolic blood pressure, medical history (prior stroke/ transient ischemic attack, hypertension, diabetes mellitus, prior coronary artery disease/myocardial infarction, atrial fib/flutter), and image data (infarction volume, pattern, and location) and stroke etiology.

† Data sets were imputed 5 times, using all covariates listed in Table 1, and results were pooled by Roubin’s rule.

**Figure S1**. Study flowchart.


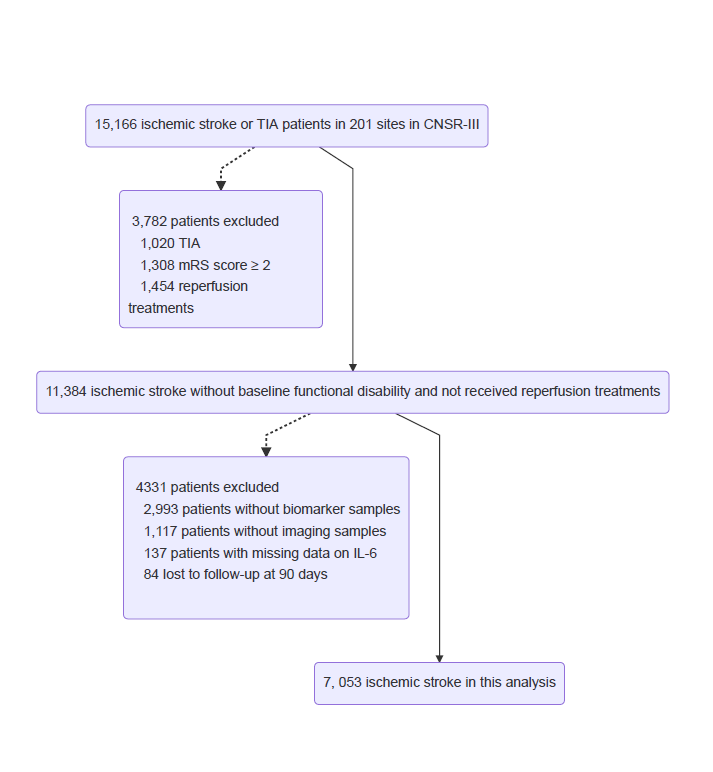


Abbreviations: IL-6 indicates Interleukin 6; mRS, modified Rankin Scale.

**Figure S2**. Distribution of IL-6 on different scales


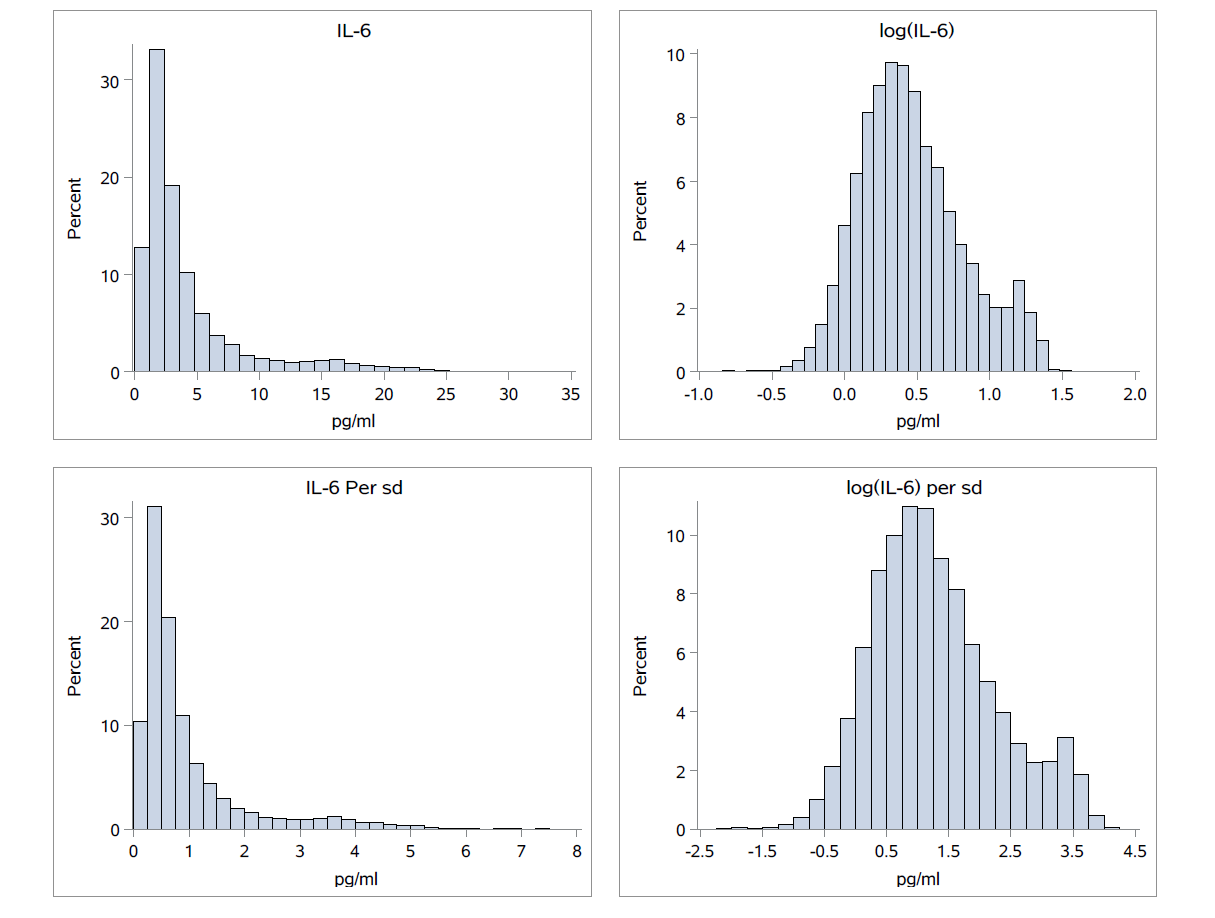


Abbreviations: IL-6, interleukin-6.
